# ROS Dependent Antifungal and Anticancer Modulations of *Piper colubrinum* Osmotin

**DOI:** 10.3390/molecules26082239

**Published:** 2021-04-13

**Authors:** Rajeswari Gopal Geetha, Sivakumar Krishnankutty Nair Chandrika, Gayathri G. Saraswathy, Asha Nair Sivakumari, Manjula Sakuntala

**Affiliations:** 1 Plant Disease Biology Laboratory, Rajiv Gandhi Centre for Biotechnology, Jagathy, Thycaud P.O., Thiruvananthapuram 695014, Kerala, India; rajeswarigopal@rgcb.res.in (R.G.G.); gayathrigs@rgcb.res.in (G.G.S.); 2 Computational Biology, Rajiv Gandhi Centre for Biotechnology, Thycaud P.O., Thiruvananthapuram 695014, Kerala, India; sivakumar@rgcb.res.in; 3 Cancer Research Program, Rajiv Gandhi Centre for Biotechnology, Thycaud P.O., Thiruvananthapuram 695014, Kerala, India; sasha@rgcb.res.in

**Keywords:** *Piper colubrinum*, osmotin, reactive oxygen species, *Phytophthora capsici*, MDAMB-231, senescence

## Abstract

Osmotin, a plant defense protein, has functional similarity to adiponectin, an insulin sensitizingsensitising hormone secreted by adipocytes. We speculated that *Piper colubrinum* Osmotin (PcOSM) could have functional roles in obesity-related cancers, especially breast cancer. Immunofluorescence assays, flow cytometry, cell cycle analysis and a senescence assay were employed to delineate the activity in MDAMB231 breast cancer cell line. PcOSM pre-treated *P. nigrum* leaves showed significant reduction in disease symptoms correlated with high ROS production. In silico analysis predicted that PcOSM has higher binding efficiency with adiponectin receptor compared to adiponectin. PcOSM was effectively taken up by MDAMB231 cancer cells which resulted in marked increase in intracellular ROS levels leading to senescence and cell cycle arrest in G2/M stage. This study provides evidence on the ROS mediated direct inhibitory activity of the plant derived osmotin protein on the phytopathogen *Phytophthora capsici*, and the additional functional roles of this plant defense protein on cancer cells through inducing ROS associated senescence. The strong leads produced from this study could be pursued further to obtain more insights into the therapeutic potential of osmotin in human cancers.

## 1. Introduction

Plants, being sessile, are constantly under attack by various pathogenic microorganisms and plants use an array of defense mechanisms to survive or retain their fitness [1]. Fungi and related oomycetes are rated as one of the most detrimental phytopathogens. In order to defend pathogens, plants use different immune strategies starting from pathogen recognition, activation of defense signal pathways and production of antifungal compounds like pathogenesis related (PR) proteins, which further restrict pathogen invasion and its replication [2,3].

Osmotin is a 24 KDa multifunctional stress responsive cationic protein, belonging to pathogenesis related-5 (PR-5) family which is accumulated in response to both biotic and abiotic stresses and is widely distributed in fruits and vegetables [4]. The presence of eight disulphide bonds stabilizes osmotin and confers resistance to pepsin digestion and heat treatment. Osmotin gene was previously isolated in our laboratory from the wild resistant pepper *P. colubrinum* and the recombinant crude osmotin protein was studied for its role as a PR protein in biotic stress tolerance and antifungal activity [5,6].

The detailed mechanism of fungal inhibition by osmotin has not been completely elucidated so far. Osmotin and osmotin-like protein (OLP) are known for their osmoprotective and antifungal roles [7]. Osmotin specifically binds with the ORE20/PHO36 transmembrane domain receptor-like protein in yeast plasma membrane and induces programmed cell death through the accumulation of ROS via RAS2/cAMP pathway [8,9]. The mammalian homologue of PHO36 receptor is Adiponectin receptor (ADIPOR1), a transmembrane receptor for adiponectin. Adiponectin is an adipokine, secreted by the adipose tissue and its levels are reduced in obesity-linked diseases such as atherosclerosis, cancer and type 2 diabetes [10]. Adiponectin mediates its effect by binding to the adiponectin receptor (ADIPOR). Adiponectin has been known to improve several diseases and therefore enhancing adiponectin signaling could be a potential therapeutic strategy for metabolism related diseases [11]. Interestingly, osmotin shares structural and functional homology with adiponectin, though there is no sequence similarity. Various studies have depicted osmotin to have exhibited adiponectin mimetic effect toward obesity, diabetes, cardiovascular and neurodegenerative disease [12,13,14,15]. The levels of adiponectin in serum have been inversely correlated with the incidence of breast cancer. Adiponectin has been shown to induce cell death and apoptosis in several breast cancer cells including triple negative MDAMB-231 cells [16,17]. Osmotin being a plant protein and an analogue of adiponectin intrigued us to understand if this could bring about any effect in this triple negative breast cancer cell line. Moreover, in light of its recent functional homology with human adiponectin, it seemed worthwhile to explore the effect of purified PcOSM in mammalian breast cancer cell lines.

Our earlier in vitro studies indicated that *P. colubrinum* osmotin exhibited significant inhibitory activity against the oomycete pathogen *Phytophthora capsici*, which urged us to study the potential in planta antifungal effects of this defense protein and its possible mechanism of action.

In the present study, therefore, we examined the functional role of recombinant purified osmotin protein in inhibiting the *Phythophthora capsici* mycelia on leaves of susceptible black pepper *P. nigrum* and its role in resistant breast cancer cell line, MDA MB-231.

## 2. Results

### 2.1. Expression and Purification of Recombinant PcOSM

**Figure 1:**
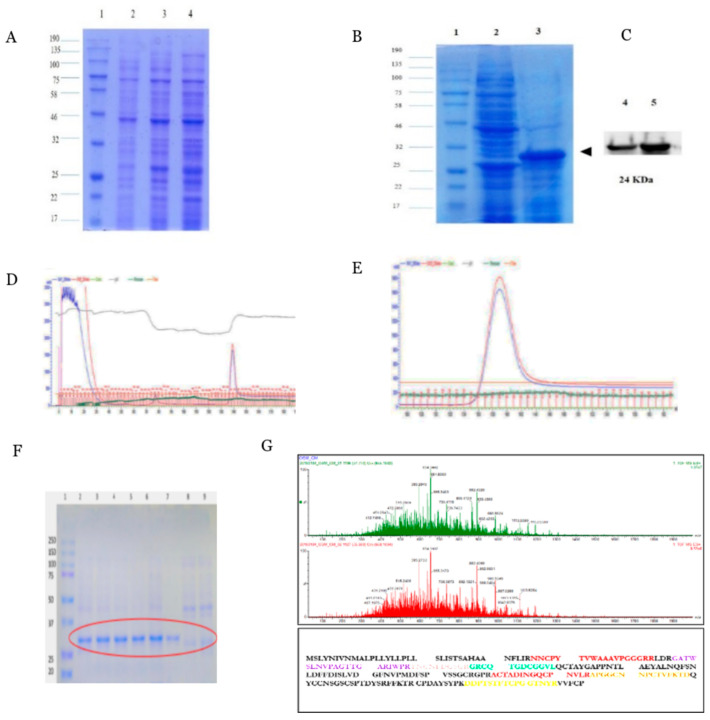
Purification of His_6_ tagged recombinant *Piper colubrinum* Osmotin in *E. coli*: (**A**) SDS-PAGE analysis of the expression of 6xHis tagged PcOSM in *E. coli* cultures expressed at 37 °C Lane 1. Molecular mass marker (NEB), (2–4)-Total protein from non-induced, induced by 1mM IPTG for 6h and 8 h. (**B**) SDS PAGE of crude protein and partially purified recombinant PcOSM protein, (**C**) Western blot analysis of His tagged PcOSM, probed with Anti-polyhistidine antibody, (**D**) and (**E**) AKTA chromatogram obtained from purification optimization. (**F**) SDS-PAGE analysis of purified elute fractions obtained from purification optimization -Lane 1: Molecular marker (NEB), lane 2-9-elute, (**G**) LC-MS/MS analysis of osmotin showing mass spectrum of osmotin and the unique peptides identified were highlighted in different colours.

Full length *Piper colubrinum* osmotin gene (PcOSM-693bp) was amplified and cloned in DH5α *E. coli* using pET100/D TOPO expression vector for heterologous expression. SDS-PAGE confirmed the presence of the recombinant protein (Figure 1A). It was found that osmotin was significantly induced at 8 h with 1 mM IPTG at 37 °C. The recombinant osmotin was found to be 27 KDa which includes the size of vector. For large scale production (2 L), BL-21 *E. coli* culture was incubated at 37 °C for 8 h, after induction with 1 mM IPTG which produced 2 mg/mL of recombinant osmotin protein (Figure 1B). The recombinant osmotin was confirmed by Western blotting using antipolyhistidine antibody (Sigma-Aldrich, St. Louis, MO, USA) (Figure 1C). Immobilized-metal affinity chromatography (IMAC) was used with an AKTA system. The AKTA chromatogram showed the high peak intensity of purified recombinant osmotin (Figure 1D,E). The fractions were eluted in elution buffer using step gradient elution and SDS-PAGE was run to confirm the purity (Figure 1F). The mature PcOSM was 230 amino acids in length with a molecular weight of 24 KDa. The protein was confirmed by LC-MS/MS (Figure 1G) which identified 9 osmotin peptides based on *P.colubrinum* osmotin protein sequence provided.

### 2.2. Inhibitory Activity of PcOSM on Hyphal Growth of Phytophthora Capsici

**Figure 2:**
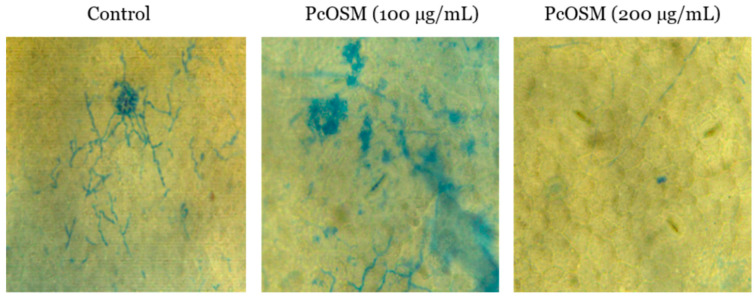
Inhibitory role of PcOSM on *Phytophtora capsici.* Control *Piper nigrum* leaf disc infected with *P. capsici*. In vivo hyphal lysis of PcOSM- *Piper nigrum* leaf infiltrated with PcOSM 100µg/mL and 20o µg/mL and infected with *P. capsici* 1 h after infiltration. The leaf discs showed significant hyphal breakage of *P. capsici* followed by inhibition of mycelial growth.

The effect of recombinant PcOSM on the hyphal growth of plant oomycete, *P. capsici* was investigated by in vivo infiltration of purified PcOSM in *Piper nigrum* leaves. Structural changes such as breakage of cell wall components, plasmolysis followed by hyphal breakage and leaching of cell components were observed on staining leaf discs with trypan blue (Figure 2). Tris buffer (pH 7.2) served as the negative control.

### 2.3. PcOSM Induced Intracellular ROS Accumulation in Leaves

**Figure 3:**
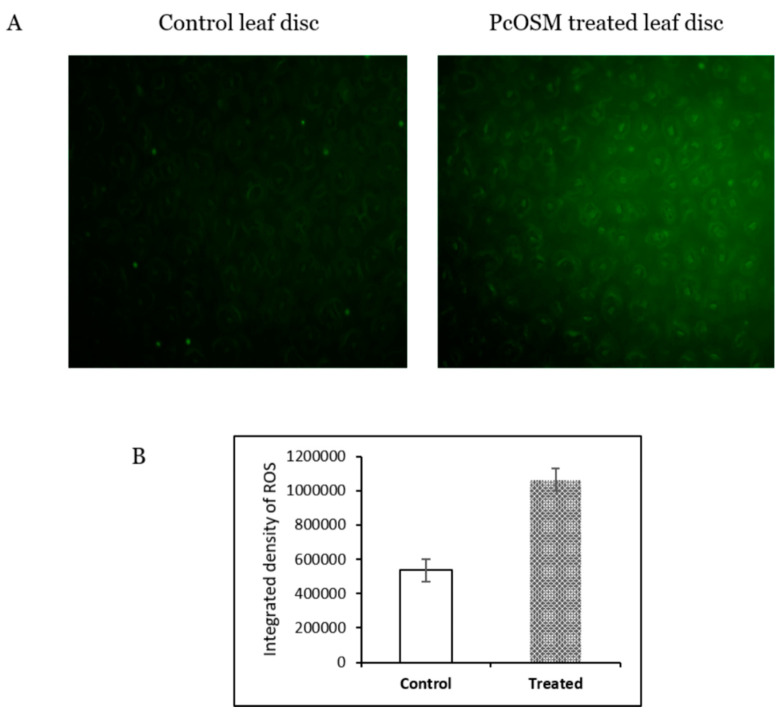
PcOSM induces ROS production in *Piper nigrum* leaves: Scale bar = 100 µm. (**A**) Fluorescence microscopic images (20×) of leaf disc stained with DCFDA to visualize ROS accumulation. (**B**) Mean values of integrated density of DCFDA staining. The asterisk indicates a significant difference (*p* < 0.05).

In our study, we observed that PcOSM induced ROS production in leaves of *P. nigrum* as observed by DCFDA fluorescence staining (Figure 3A). There was a significant increase in fluorescence due to ROS accumulation in PcOSM-treated leaf disc (Figure 3B).

### 2.4. Molecular Docking and Simulation

**Figure 4:**
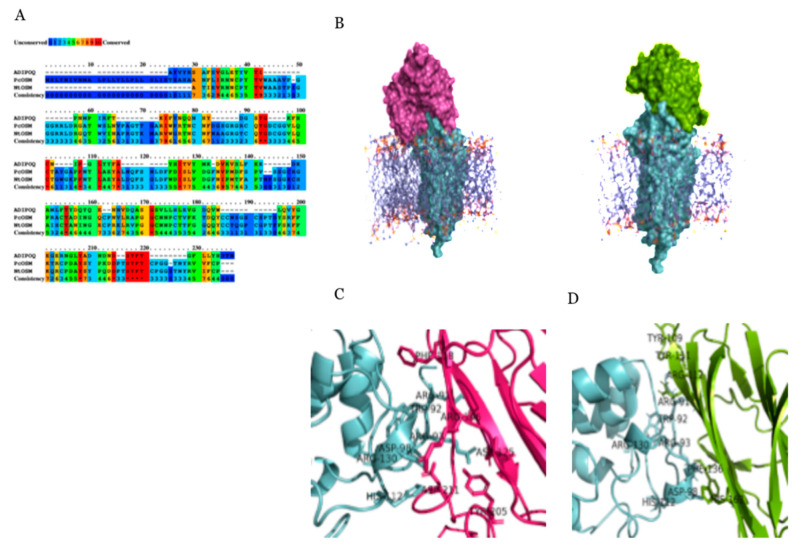
Molecular docking studies: (**A**) The multiple alignments of amino acid sequences of ADIPOQ, PcOSM, NtOSM compiled with PRALINE. Coloured blocks indicate conserved regions. (**B**) The helixes of ADIPOR1 are embedded in lipid bilayer membrane subjected to supervised docking with OSM (purple) and ADIPOQ (green) restricting their docking grid confined to intra-cellular loops of ADIPOR1. The lowest energy docking solutions were submitted to MD simulation for structural refinement. (**C**) Representative snapshot for free energy minima of ADIPOR1/OSM and (**D**) ADIPOR1/ADIPOQ complexes from simulations showing functional residues contributing highly towards effective binding at the interaction interface.

Multiple alignments with the protein sequences from ADIPOQ, PcOSM, NtOSM was constructed employing the PRALINE server using progressive alignment strategy. Comparison of sequences revealed that OSM remained highly conserved between species through the entire length of protein (Figure 4A), it is very likely to have similar protein folding and conserved function for each of the polarity complexes. Overall, the multiple alignments revealed significant <%> sequence identity between the sequences across the global alignment. Patchdock a geometry-based molecular docking algorithm was used to simulate docking of ADIPOR1 with PcOSM and ADIPOQ. Unlike blind docking, where the docking software seeks to find the best orientation of the ligand in a grid covering the entire receptor protein, supervised docking was performed with the grid confined to intra-cellular loops of ADIPOR1 by embedding the transmembrane helixes in a membrane bilayer to improve the enrichment of docked conformers (Figure 4B). Both the proteins possess a similar binding mode with ADIPOR1 and have the conserved interacting residues of their domain. The presence of polar charged residues (i.e., 91ARG, 93ARG, 98ASP, 112HIS, 130ARG in ADIPOR1) stabilizes the interaction with PcOSM and ADIPOQ and play an essential role in their recognition. Conserved polar charged interacting residues of PcOSM (i.e., ASP192, LYS198, ARG200, ASP203, ASP210, ASP211) and ADIPOQ (i.e., ARG112, HIS163) interacts with ADIPOR1, thereby improving the electrostatic environment of the protein-protein interaction (Figure 4C). Furthermore, the closeness of aromatic residues (i.e., 109TYR, 111TYR, 136PHE, 163HIS in ADIPOQ and TYR181, PHE196, PHE197, TYR205, TYR207 in PcOSM) stabilizes the interaction with 92TRP, 112HIS residues of ADIPOR1 between the proteins and might play a significant part in stacking interactions (Figure 4D). Interaction energies were evaluated by Lennard-Jones potential and Coulomb’s Law (parameters from the GROMOS96 53a6 force field). We found that the interaction energies were—14699.563 kcal/mol and—17826.805 kcal/mol for the ADIPOR1/ADIPOQ and the ADIPOR1/OSM complexes, respectively. ADIPOR1 binds extensively with PcOSM which buries a total of 2584 Å solvent accessible surface area (SASA), while it buries a total of 1947Å solvent accessible surface area with ADIPOQ at the interaction interface.

### 2.5. PcOSM Showed no Cytotoxicity in MDA MB231

**Figure 5:**
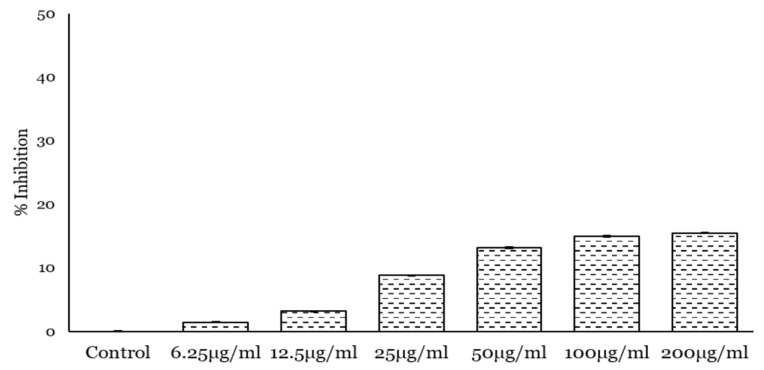
Cytotoxicity effect on MDA MB231 cells treated with PcOSM for 72 h measured by MTT assay. Results are reported as percentage of cell death. The bar shows mean ± Standard deviation of triplicates. Statistical significance of the treated and untreated cells were analyzed using one way ANNOVA. PcOSM treated cells were significantly different from control cells (*p* < 0.001).

To investigate the effect of PcOSM on breast cancer cells, we choose a triple negative breast cancer cell line-MDA MB231. Initially the antiproliferative effect was monitored by MTT assay. Varying concentrations of PcOSM from 200 µg to 6.25 µg/mL were added to cells and treated for 24, 48 and 72 h. PcOSM did not cause any cell death as revealed by the MTT assay (Figure 5), but instead there was a marked alteration of cell morphology. In contrast to control cells, PcOSM-treated cells have lost its definite structure with an irregularity in the cell membrane structure as visualized by bright field microscopy.

### 2.6. ADIPOR1 Localized on Cell Membrane and Showed Co-Localization with PcOSM–ADIPOR1

**Figure 6:**
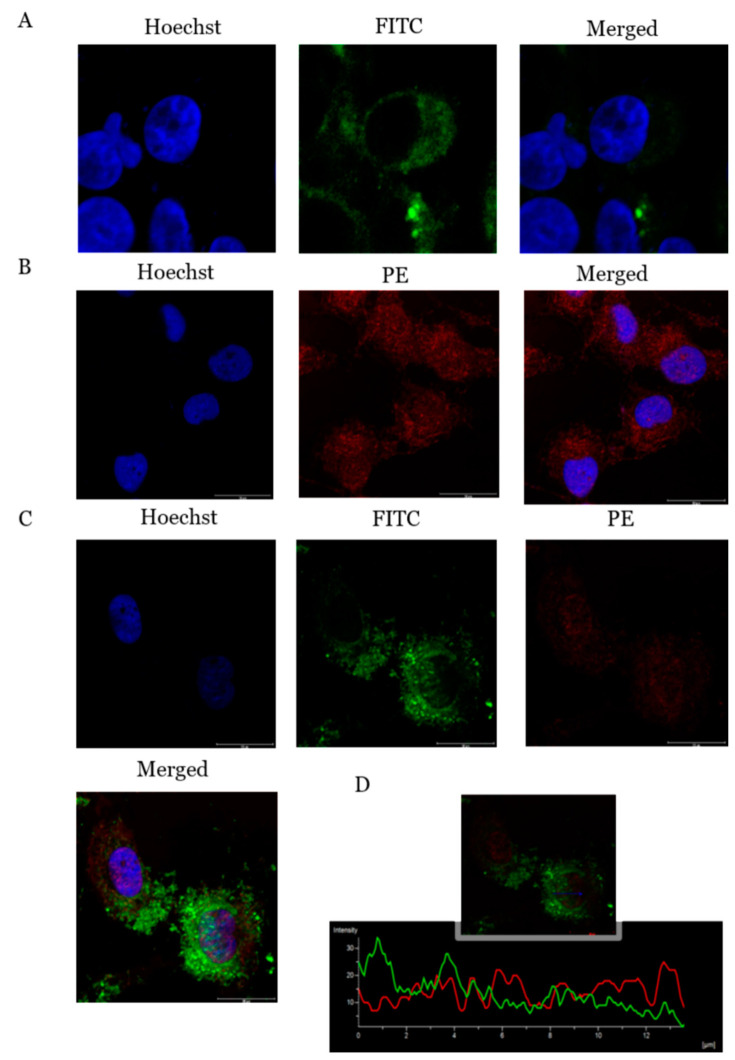
Immunofluorescence assays: (**A**) MDA MB231 cells stained with FITC labelled secondary antibody for ADIPOR1 (green colour) and Hoechst 33342 to stain the nuclei (blue colour). (**B**) Cells were stained with Phycoerythrin labelled secondary antibody for histidine tagged PcOSM. (**C**) C0-immunofluorescence assay for co-localization of PCOSM and ADIPOR1, cells were co stained with secondary antibodies for ADIPOR1 and PcOSM. (**D**) Intensity profile of co-localization of ADIPO-R1 and PcOSM of an area indicated in the image analysed by NIS Elements imaging software.

Immunofluorescence was conducted to characterize the presence and localization of ADIPOR1 (Figure 6A) as well as PcOSM (Figure 6B) in MDAMB231 cells. The ADIPOR1 receptor was found to be localized in the cell membrane. Adiponectin specific receptors are expressed on several breast cancer cell lines especially MDAMB231 [18]. PcOSM immunofluorescence assay revealed that osmotin localized on the cell membrane an at 72 h of treatment could bring about a change in the morphology of the cell membrane with the cells attaining a rounded morphology. It was also observed that PcOSM showed internalization into the cytoplasm and nuclear regions also.

Co-immunofluorescence assay confirmed the interaction of PcOSM with ADIPOR1 (Figure 6C). Unlikely to the untreated MDAMB231 cells, where the expression of ADIPOR1 was localized in the cell membrane, the PcOSM-treated cells showed the co-localization of both ADIPOR1 and PcOSM in the cytoplasmic as well as the nuclear region. There was an increase in the fluorescence of ADIPOR1-PcOSM in the nuclear region compared to that of cytoplasmic region; the Pearson correlation of 0.6971, Mander’s overlap of 0.7514 and co-localization coefficient of 0.7669.

### 2.7. PcOSM Induced Disintegration of Cytoskeletal Elements

**Figure 7:**
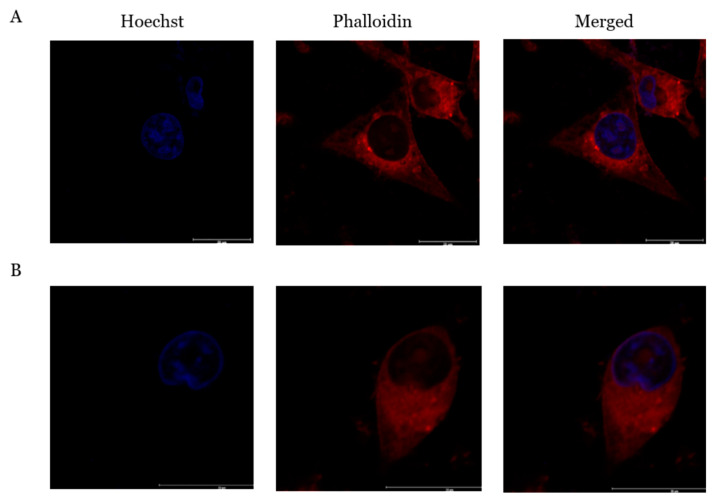
Phalloidin staining for cytoskeleton: (**A**) Untreated MDA MB231 cells stained with rhodamine- Phalloidin showing rigid cytoskeleton. (**B**) PcOSM treated MDAMB-231 cells showing cytoskeletal changes.

Immunofluorescence assay revealed morphological changes suggesting that PcOSM might affect cytoskeleton. Since the cellular morphology showed characteristic changes and cells showed induced stress, we hypothesized that the cytoskeletal protein might be altered. Therefore, the cytoskeletal integrity was assessed by phalloidin staining. To study the changes in actin distribution, rhodamine–phalloidin staining was conducted. PcOSM disintegrated filamentous actin. In control cells, (Figure 7A), F-actin showed a network extending continuously in the cytoplasm. The experiments revealed that PcOSM-treated cells had lost its cell integrity. There was significant cytoskeletal damage in PcOSM-treated MDA MB231 cells (Figure 7B).

### 2.8. PcOSM Increased Intracellular Reactive Oxygen Species (ROS) Levels

ROS signaling molecules, play significant roles in signal transduction and has found to be critical for maintenance of cellular homeostasis. ROS can be an efficient therapeutic tool in anticancer therapies, based on oxidative damage as a result of its accumulation. To understand if a similar role of PcOSM ensues in cancer cells, we checked for the ROS alteration in MDA MB231 cells. Increasing ROS levels were detected using a cell permeable fluorogenic probe dichlorofluorescein diacetate (DCFDA) and confirmed by confocal microscopy and flow cytometry.

**Figure 8:**
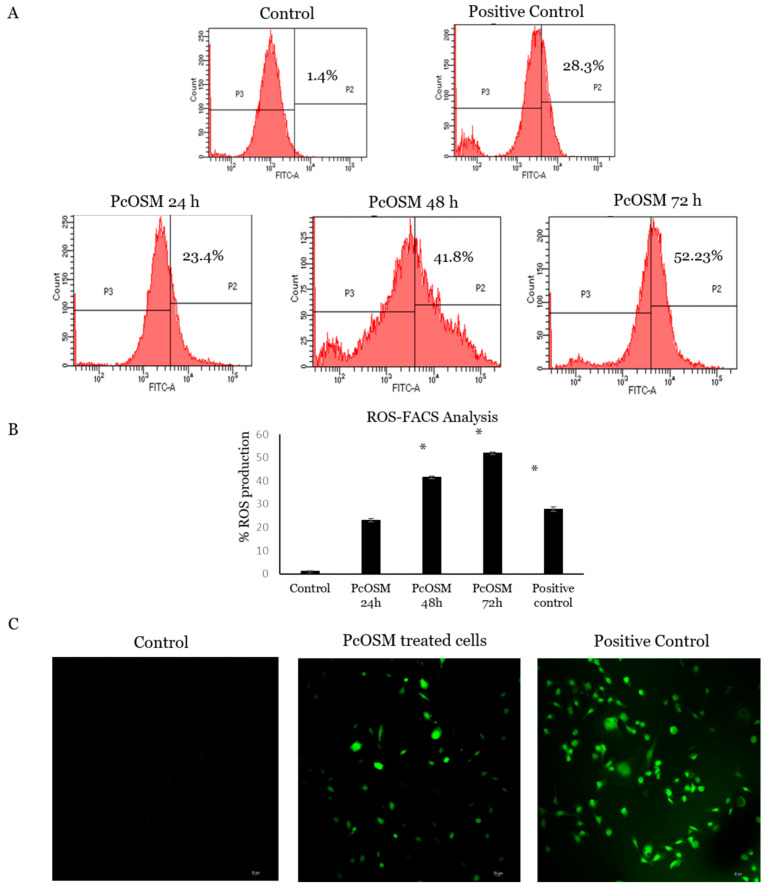
PcOSM induced intracellular reactive oxygen species accumulation: (**A**) Flow cytometry analysis of reactive oxygen species (ROS) production in control cells, positive control and PcOSM treated MDAMB231 cells for 24, 48 and 72 h. (**B**) Representative histogram of DCFDA staining of cells treated with PcOSM. Columns indicate mean ± SD of three experiments. * *p* < 0.05 vs. Control. (**C**) Confocal microscopy images (20X) showing ROS production in positive control and PcOSM after 72 h of treatment.

MDAMB231 cells were treated with PcOSM for 24, 48 and 72 h and stained with DCFDA to investigate the accumulation of intracellular ROS levels and analyzed using flow cytometry and confocal microscopy. The flow cytometry results revealed that PcOSM induced tremendous accumulation of ROS in a time dependent manner. There was a significant increase in ROS accumulation in PcOSM-treated cells compared to both positive control and untreated cells (Figure 8A). Confocal microscopy on 72 h treated cells and showed a significant increase in accumulation of ROS in treated cells as well as tert-butyl hydroperoxide (TBHP) treated cells in comparison with the untreated cells (Figure 8B). These results implicated that PcOSM could induce intracellular ROS in MDAMB231 cells.

### 2.9. PcOSM Induced Senescence in MDAMB231 Cells

**Figure 9:**
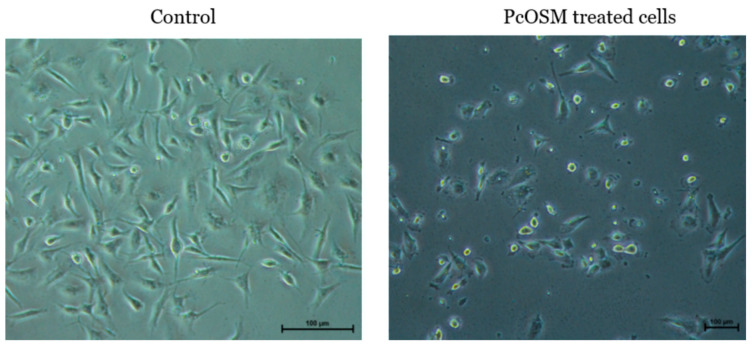
PcOSM induced cellular senescence in MDAMB231 cells: Senescence associated beta galactosidase assay (SA-β-Gal) staining of cells indicated concentration of PcOSM after 72 h. Cells were fixed and stained with fresh SA-β-Gal. PcOSM treated cells exhibited blue colour indicating senescent cells.

In our initial observation, PcOSM-treated MDAMB231 cells had phenotypes characterized by enlarged cellular size and nuclei with flattened morphology and changes in cytoskeletal structures which could strongly indicate senescence phenotype. Senescence-associated β-galactosidase assay, a sensitive method for the detection of cellular senescence was carried out to identify if senescence occurred. An increase in the level of lysosomal mass leading to the production of higher level of β-galactosidase activity was observed in PcOSM-treated MDAMB231 cells after 72 h (Figure 9). MDAMB231 cells showed were stained with blue color as observed in bright field microscopy which in turn denotes the accumulation of lysosomes.

### 2.10. PcOSM Induced Cell Cycle Arrest in MDAMB231 Cells

**Figure 10:**
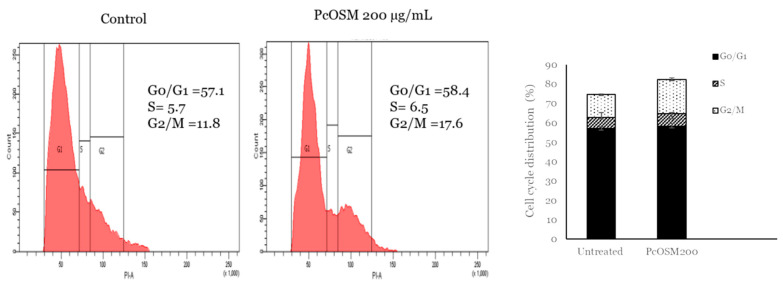
PcOSM induced cell cycle arrest: MDA MB231 cells were treated with PcOSM (200 µg/mL) for 72 h. Cell cycle analysis was performed using PI staining. Percentage of cell cycle distributions are shown. The results indicate the mean ± SD of three independent experiments.

PcOSM neither induced cell death nor apoptosis but there was a significant effect on cell morphology and ROS accumulation. Hence, we decided to analyze the effect on cell cycle. MDAMB231 cells were treated with PcOSM for 72 h and stained with PI to investigate cell cycle arrest by flow cytometry. The cell cycle showed an increase in G2/M phase when stimulated with PcOSM (200 µg/mL) (Figure 10).

## 3. Discussion

Osmotin is a plant pathogenesis-related protein with proven pathogen defense role in plants [19]. Recently this protein has attracted a lot of attention due to its functional similarity with adiponectin and hence it has implications in obesity related diseases such as cardiovascular and neurodegenerative diseases. Osmotin was found to be neuroprotective and prevented amyloid beta-induced neurodegeneration [20]. Osmotin acts as an adiponectin agonist in obesity and type 2 diabetes and also exhibited antiatherosclerotic effects when administered intravenously in experimental rats [21,22]. Similar to adiponectin, osmotin also exerted an antiinflammatory role in murine colitis [23].

*Piper colubrinum* is a wild species of pepper that is distantly related to cultivated black pepper (*Piper nigrum*). The plant is highly resistant to many microbial pathogens infecting cultivated pepper including *Phytophthora capsici* that causes the much dreaded ‘quick wilt’ in black pepper plantations. Earlier studies in our laboratory have established the potential of wild *P. colubrinum* as a rich repository of defense genes. Recombinant crude protein from *P. colubrinum* exhibited antifungal activity against *Phytophthora capsici* in vitro [6]. In this paper we report the effective purification and renaturation of recombinant PcOSM by slow dialysis against oxidized and reduced glutathione, which resulted in the renaturation and subsequent refolding of functionally active osmotin protein. The purified protein showed strong inhibitory activity on *P. capsici* in vivo and induced significant ROS production on leaves of susceptible black pepper, which led us to hypothesize that the fungal toxicity exhibited by osmotin presumably coincides with increased ROS production. There are no reports so far implicating the role of ROS in the activity of osmotin on plants. Moreover, the protective effect of osmotin treatment on leaves through its involvement in ROS production suggests a possible role of osmotin as a priming agent. Defense priming is implicated in plant defense through ROS-mediated stimulation of hypersensitive response (HR) and innate immunity [24,25].

In *Saccharomyces cerevisiae,* osmotin act as an antifungal cytotoxic compound which induces rapid cell death [26]. Several antifungal proteins exhibit membrane disruptive effects, increase membrane permeability, cause morphological changes and also enhance ROS production resulting in fungal cell death [27,28]. In a previous report by Narasimhan et al. osmotin-induced apoptosis in yeast cells was correlated to intracellular ROS generation through Ras/cAMP pathway. It was found that ROS act as effectors of osmotin-induced cell death.

Osmotin is a structural and functional homologue of adiponectin, a hormone secreted by adipocytes and domain I of osmotin is the structural homolog of the β-barrel domain of adiponectin [29]. PHO-36 is a seven transmembrane receptor found on the yeast cell wall and this receptor is required for the activity of osmotin in yeast. PHO36 mimics ADIPOR1-1 and, consequently, osmotin binds with Adiponectin receptor-1 (ADIPOR1) and mediates similar mechanisms that of adiponectin. The levels of adiponectin are inversely correlated with the incidence of breast cancer [30]. Interestingly, the correlation between adiponectin and breast cancer has found to be prominent in ER/PR-negative breast cancer [16]. Adiponectin was reported to inhibit growth and enhance apoptosis in ER/PR-negative cell lines [18]. We demonstrated the functional role of PcOSM on triple negative breast cancer cell line MDAMB231 and we noticed that PcOSM caused morphological changes on cells without cytotoxicity. Osmotin is naturally present in all fruits and vegetables and induce stress tolerance in them. It is an inducible protein which is significantly overexpressed under conditions of abiotic and biotic stress. However, at basal level it acts as a house keeping gene [19]. To the best of our knowledge, no reports of Osmotin toxicity in plants have been reported so far. Osmotin was observed to be non-toxic in mammalian systems, as it had no adverse effect on Hek 293T cells (human embryonal kidney cells) even at higher concentrations of 500 µg/mL and did not exhibit any hemolytic activity [31]. Even though PcOSM did not cause cytotoxicity, the effect on membrane was very significant. Osmotin induced membrane permeabilization in *Trichoderma longibrachiatum* [32]. However, osmotin induces growth inhibition in several fungus through plasma membrane permeabilization and dissipation of the membrane potential [33]. Similar to these reports, we also observed plasma membrane permeabilization in MDA MB231 cell line. PcOSM was found to affect the cell membrane integrity and showed nuclear localization as revealed by the immunofluorescence assay. Various studies have proven cell membrane to be a major target of anticancer drug action [34]. The mechanism of action of cell membrane acting protein like thionin, small protein with 3–4 cysteine residues is attributable to a cellular response that involves stimulation of Ca^2+^ influx coupled with membrane depolarization. This can further lead to the activation of phospholipase A2, membrane alteration and finally cell death [35]. The activity of plant proteins depends on their characteristics as well as the target membrane [36]. Cysteine rich proteins have the potential to induce membrane depolarization, and osmotin, being a cationic protein, possesses 16 cysteine residues. Double fluorescence staining revealed that both PcOSM and ADIPOR1 co-localized and there was an increase in fluorescence in the nuclear region. We presume that cell membrane penetration by PcOSM may be accelerated by ADIPOR-1. To ascertain the loss of cell membrane integrity by PcOSM analysis of actin cytoskeleton of MDAMB231 cells was achieved using rhodamine-phalloidin (for F-actin) staining. Untreated cells had dense and dynamic mesh-work of long filamentous actin whereas the actin filaments of PcOSM-treated cells were disorganized and disrupted. These observations suggest that PcOSM caused the breakdown of actin thereby causing marked cytoskeletal changes in the MDAMB231 cells compared to the untreated cells. Osmotin has been reported to regulate cytoskeletal organization and mediates programmed cell death under cold stress in olive trees [37]. However, to our knowledge we demonstrated for the first time the effect of osmotin on MDA MB231 breast cancer cell line. To further understand the mechanism of action of PcOSM, ROS accumulation was analyzed through DCFDA staining. Many plant-derived compounds have been reported to induce ROS accumulation in breast cancer cell line [38]. Osmotin is a stable defense plant protein and is widely distributed in fruits and vegetables and protects plants from pathogens.

PcOSM induced significant ROS generation in breast cancer cell line higher than that of TBHP and ROS induced morphological changes in MDA MB231 cells leading to senescence. Flow cytometry analysis showed a time dependent effect of PcOSM in ROS generation. There was an increase in fluorescence in the 72 h PcOSM-treated cells showing an increase in cytosolic ROS generation. In the previous experiment, it was shown that ROS accumulation lead to *P. capsici* hyphal breakage and release of contents. Similar to the effect in *P. capsici*, here we also observed ROS accumulation in MDAMB231 cells. ROS can cause damage to lipids, proteins and carbohydrates thus leading to loss of cellular integrity. The strategy of many cancer therapeutics agent necessarily stands to exuberantly intensify the intracellular ROS leading to irreparable damage and finally tumor cell apoptosis [39]. Numerous anticancer agents and ionizing radiation destroy tumor cells by generating ROS.

Even though ROS promotes tumorigenesis, angiogenesis and metastasis, excessive accumulation of ROS can induce cell death [40]. It can act as a secondary messenger and trigger oxidative DNA damage, modulate several cell signaling pathways leading to senescence. Cellular senescence is a condition of irreversible cell cycle arrest and is triggered by numerous stresses mainly oxidative stress, DNA damage and telomere shortening [41]. Induction of cellular senescence was investigated by senescence associated beta galactosidase assay (SA-β-gal) that could also display lysosomal dysfunction. SA-β-gal is a widely used biomarker for determining the β-galactosidase activity in cells. PcOSM induced senescence in MDAMB231 cells through an increased level of β-galactosidase activity, as observed by the blue color in the cells. PcOSM-treated MDAMB231 cells showed irregular nuclei and morphological changes and also induced ROS accumulation after 72 h of treatment further leading to senescence. Cellular senescence leads to irreversible cell cycle arrest in response to various cellular stresses. Irreparable DNA damage interrupts cell cycle causing G2 arrest followed by mitotic bypass into G1phase and culminates in cellular senescence [42]. Several natural dietary compounds selectively target cancer cells because increased ROS levels can cause oxidative stress threshold sooner in cancer cells compared to normal cells [43] and the oxidative stress can act as a determining factor of cellular senescence. Therapy induced senescence is a new field in cancer therapeutics and induce cytostatic state in tumor cells [44]. We also observed cytostatic state in PcOSM-treated MDAMB231 cells suggesting a senescence inducing role by the overexpressed recombinant *Piper colubrinum* osmotin.

## 4. Materials and Methods

### 4.1. Plants and Maintenance

Cuttings of *P. colubrinum* were rooted in sterile soilrite mixture (peat moss:vermiculite:perlite, 1:1:1, *v*/*v*/*v*, Keltech Energies Ltd., Bangalore, India). Rooted plants of uniform age were maintained in the growth chamber (Conviron CMP6010) with the temperature adjusted to 24 °C and humidity of 70% and under a 16/8 h light/dark regime in the Plant Tissue Culture facility of Rajiv Gandhi Centre for Biotechnology. Young leaves from 2-month-old cuttings were used for RNA isolation and cloning of osmotin gene.

Cuttings of *Piper nigrum* were similarly maintained under controlled conditions in the growth chamber and young leaves from 3-month-old cuttings were selected for pathogen infection assays.

### 4.2. Cloning and Expression of P. colubrinum Osmotin in E. coli

RNA isolation and RT PCR were performed based on earlier protocol [6] with modifications. Full length osmotin gene was cloned from young leaves (3rd leaves) of *P.colubrinum* plants using the primers **CACCATGTCACTATACAATATAGTAAACATGGCC** and **TGGGCAGAAGACAACTCTGT** as forward and reverse primers of *Piper colubinum* osmotin gene (GeneBank accession: EU 271754.1). The full length osmotin gene was cloned in pET100/D TOPO (Champion™, pET Directional TOPO expression kit, Invitrogen) for the expression of recombinant osmotin protein [45]. PCR was performed in an Eppendorf thermocycler with 35 cycles of amplification under the following conditions −95 °C for 30 s, 55 °C for 30 s and 72 °C for 1 min using High fidelity Phusion DNA polymerase (Invitrogen). The recombinant plasmid was transformed into the bacterial strain DH5α using standard procedures and colonies were verified by sequencing both the strands of PcOSM using an automated ABI sequencer with Big Dye Terminator v3.1Cycle sequencing kit (Thermo Fisher Scientific, Waltham, MA, USA).

### 4.3. IPTG Induction and Purification of Recombinant PcOSM

BL-21 competent cells were transformed with pET100/D-TOPO containing recombinant PcOSM. For protein expression, BL 21 *E. coli* cells carrying recombinant PcOSM were cultured overnight at 37 °C in 5 mL Luria-Bertani (LB) broth containing 100 µg/mL ampicillin with vigorous shaking (220 rpm) until the OD (600 nm) reached 0.6–0.8. IPTG Induction was carried out with a concentration of 1 mM for 8 h and afterwards the cells were pelleted at 4000× *g* at 4 °C for 20 min. Osmotin being a vacuolar protein requires denaturing buffer (0.1 M Tris HCl and 6 M Urea, pH 8.0) for extraction of protein from pellet. The pellet was suspended in urea buffer, mixed well and kept in ice for 30 min, and then the slurry was kept under constant shaking for 45 min, to completely dissolve the pellet. The cellular debris was removed by centrifugation at 10,000× *g* at 4 °C for 25 min. The supernatant was collected and the step was repeated twice to solubilize the protein completely.

### 4.4. SDS PAGE and Western Blot Analysis

The supernatant was pooled the total protein was analyzed by 12% sodium dodecyl sulphate polyacrylamide (SDS PAGE). Blue prestained protein standard (NEB#p7718) was used as protein marker for SDS-PAGE and then visualized by Coomassie brilliant blue staining. The separated recombinant PcOSM protein was transferred to a polyvinlylidene (PVDF) membrane using mini trans blot cell (Bio-rad) for 1 h at 100 V. The membrane was incubated for 1 h at room temperature in a blocking buffer (5% bovine serum albumin (BSA) in 1 X Tris buffered salina-0.1% tween 20) and then probed with anti-poly Histidine antibody (diluted 1:5000; Sigma-Aldrich) overnight at 4 °C [46]. The membrane was washed with wash buffer 3 times for 10 min and incubated with horseradish peroxidase conjugated anti-mouse IgG secondary antibody (diluted 1:5000; Sigma-Aldrich) for 1 h at room temperature. Immunoreactive bands were visualized with an enhanced chemiluminescence substrate (Biorad).

### 4.5. Protein Purification

Protein purification was performed using high-performance immobilized-metal ion affinity chromatographic (IMAC). The IMAC column containing Ni-NTA agarose beads was equilibrated in a buffer containing 0.1 M Tris HCl and 6 M Urea at pH 8.0 and subsequent wash was performed using buffer (0.1 M Tris HCl, 6 M Urea and 20 mM Imidazole, pH 8.0). The recombinant protein was eluted in elution buffer (0.1 M Tris HCl, 6 M Urea and 200 mM Imidazole, pH 8.0) by step gradient elution in AKTA system. The protein purification step was conducted in Centre for cellular and Molecular platforms (CCAMP), (Bangalore, India). Protein fractions were pooled and subjected to dialysis using 10 KDa MWCO membrane into refolding buffer (20 mM Tris HCl pH 7.2, 500 mM NaCl, 10 mM reduced glutathione, 1 mM oxidized glutathione at 4 °C. The dialyzed sample was further dialyzed against Milliq water at 4 °C to slowly remove denaturants [47]. The dialyzed protein was concentrated using Amicon concentrator (10 KDaMWCO) and protein concentration was measured. The identity of purified recombinant osmotin (PcOSM) protein was confirmed by liquid chromatography tandem mass spectrometry (LC-MS/MS) analysis.

### 4.6. Liquid Chromatography Tandem Mass Spectrometry

The tryptic peptides were separated using a nanoACQUITY UPLC^®^ chromatographic system (Waters, Manchester, UK). Instrument control and data processing was done with MassLynx4.1 SCN781 software. The peptides were separated by reversed-phase chromatography. MS analysis of eluting peptides was carried out on a SYNAPT^®^ G2 High Definition MS™ System (HDMS^E^ System, Waters). The instrument settings were: nano-ESI capillary voltage 3.5 KV, sample cone 40 V, extraction cone 6 V, IMS gas (N_2_) flow–90 (mL/min). All analyses were performed in positive mode ESI using a NanoLockSpray™ source.

### 4.7. LC-MS/MS Data Analysis

The acquired ion mobility enhanced MSE spectrum was analyzed using Progenesis QI for Proteomics V3.0 (Non Linear Dynamics, Waters) for protein identification. Data processing included lock mass correction post acquisition. Processing parameters for Progenesis were set as follows: noise reduction thresholds for low energy scan and high energy scans were calculated automatically by using ion accounting workflow in the software. The protein identifications were obtained by searching against a custom database having pathogenesis related proteins from *Piper colubrinum*, *Calotropis procera*, *Nicotiana tabacum*, *Actinidia deliciose* and *Arabidopsis thaliana* from UniProt. During database search, the protein false positive rate was set to 4%. The parameters for protein identification was made in such a way that a peptide was required to have at least one fragment ion match, a protein was required to have at least three fragment ion matches and a protein was required to have at least one peptide match for identification. Oxidation of methionine was selected as variable modification and cysteine carbamidomethylation was selected as a fixed modification. Trypsin was chosen as the enzyme used with a specificity of one missed cleavage.

### 4.8. Fungal Growth Inhibition Bioassays

#### 4.8.1. Infiltration Method

The purified and refolded PcOSM was assayed for its ability to inhibit mycelial growth of *Phytophthora capsici* oomycete by in vivo *leaf* infiltration method. In total, 100 µL of PcOSM in 20 mM Tris Cl at concentrations 100 and 200 µg/mL were infiltrated into the basal side of *Piper nigrum* leaves. Leaves were previously surface sterilized using 0.1% HgCl_2_ followed by rinsing in sterile water. Infiltration was carried out using needless syringe [48].

#### 4.8.2. Infection Assay and Trypan Blue Staining

After 1 h of PcOSM infiltration, infection was initiated by placing a *P. capsici* plug isolated from a fresh plate of *P. capsici* maintained in sterile potato dextrose agar (PDA) medium, following established protocol [6]. The plugs were placed on the abaxial surface of the leaves and leaves were placed in a moist chamber to obtain high relative humidity. After 24 h of incubation, leaf discs were separated for *P. capsici* staining.

For Trypan blue staining of fungal hyphae, leaf discs were placed in a 12-well plate containing solution A (Acetic acid: ethanol (1:3 *v*/*v*) and incubated at room temperature in a rocker overnight. After incubation, solution A was removed and solution B (acetic acid:ethanol:glycerol (1:5:1) was added and incubated for 3 h to remove chlorophyll followed by overnight staining in 0.1% Trypan blue. After destaining with 60% glycerol for 3–6 h, the leaf discs were mounted and visualized in a Nikon Eclipse Ni microscope, Japan.

### 4.9. Measurement of ROS Production

The endogenous ROS production in *P. nigrum* leaves in response to osmotin treatment was visualized by DCFDA staining following earlier protocol [49]. Briefly, the leaves were infiltrated with PcOSM (200 µg/mL). Leaf discs were submerged in 50 µM DCFDA solution in 10 mM Tris Cl (pH 7.5) for 10 min. The leaf discs were rinsed with PBS buffer and mounted with PBS buffer. The ROS accumulation was visualized as green fluorescence on osmotin infiltrated leaf discs with a Nikon Eclipse Ni microscope, Japan, of an excitation 488 nm and 525 nm emission. The integrated density was measured using ImageJ software.

### 4.10. Docking Studies

Multiple alignments of adiponectin (ADIPOQ), *Piper colubrinum* osmotin (PcOSM), *Nicotiana tabacum* osmotin (NtOSM) were performed using the PRALINE server using progressive alignment strategy [50]. The solved crystal structures of ADIPOR1 (3WXV), ADIPOQ (4DOU) and OSM (1PCV) were retrieved from the Protein Data Bank and employed in docking and molecular simulation studies. PatchDock web server was used to identify interactions between the complexes [51]. GROMACS v5.0.7 was further applied to simulate each complex under dynamic conditions using classical molecular dynamics (MD) theory [52]. A set of protocols in Gromacs was utilized to analyze MD trajectories.

### 4.11. MDA MB231Proliferation Assay

MDA MB231 cell lines were obtained from American Type Culture Collection (ATCC) and were cultured according to standard mammalian cell culture protocols. MDA MB231, a highly metastatic triple negative breast adenocarcinoma cell line, was seeded on to 96 well plate (5 × 10^3^ cells/100 µL/well) and incubated at 37 °C in 5% CO_2_ for 24 h in Dulbecco’s modifies Eagle’s medium (DMEM) supplemented with 10% fetal bovine serum (FBS) and 1% penicillin-streptomycin (Invitrogen, MA, USA). Cells were treated with the indicated concentration of PcOSM in fresh media. The plates were incubated separately for 24, 48 and 72 h. Then 10 µL of MTT dye was added to each well. After 4 h of incubation, the amount of formazan product was determined by reading the absorbance at 570 nm using a Tecan microplate reader.

### 4.12. Immunofluorescence Staining

MDA MB231 cells (1.5 × 10^4^/well) were grown on a coverslip in a 12 well plate and treated with the indicated concentration of PcOSM for 72 h. After treatment cells were washed with 1 X PBS and fixed with methanol: acetone (1:1) and incubated at −20 °C for 20 min blocked in PBS containing 3% bovine serum albumin (BSA) for 30 min at room temperature. Cells were then incubated with primary antibody overnight at 4 °C followed by anti-rabbit IgG-PE/anti-mouse IgG-FITC and counterstained with Hoechst 33342 for 10 min [53]. After washing with PBS samples were mounted on slides and imaged using a laser scanning confocal microscopy (Nikon Inverted Microscope Eclipse Ti-E, Nikon A1R, Japan) with a 60× objective lens.

For F-actin visualization, rhodamine-phalloidin (Abcam) was used. Cells were washed with 1XPBS, blocked with 3% BSA and stained with rhodamine-phalloidin in blocking solution for 15–20 min, washed with PBS and counterstained with Hoechst 33342 (Sigma-Aldrich, USA). The samples were washed with PBS, mounted in buffered glycerol (0.1 M, pH 9.5) and viewed microscopically [54]. The images were scanned under 60× objective.

For co-immunofluorescence after fixation and blocking with 3% BSA, PcOSM-treated cells were incubated with anti-adiponectin receptor antibody for 1.5 h followed by anti-his antibody for 1.5 h. The secondary antibodies-Goat anti-mouse IgG-PE and mouse anti goat IgG-FITC. Nuclei were counterstained by Hoechst and samples were imaged using a laser scanning confocal microscopy and co-localization was quantitatively assessed by Pearson correlation and co-localization coefficient using NIS elements software.

### 4.13. ROS Detection Assay–DCFDA Fluorescent Microscopy and Flow Cytometry Assay

To detect the accumulation ROS in MDAMB 231 cells following PcOSM treatment, DCFDA cellular ROS detection assay was carried out following the instructions in the manual provided by the kit (Abcam). Briefly, MDA MB231 cells were grown on a coverslip and treated with the indicated concentration of PcOSM for 72 h for live cell imaging. The cells were washed with 1X buffer supplied by the kit and stained with 20 µM DCFDA for 30 min at 37 °C. The cells were washed with 1X buffer and imaged using confocal microscopy. Low light conditions were avoided to prevent photo bleaching and photo oxidation.

For flow cytometry analysis. MDA MB231 cells (1.5 × 10^6^) were grown in a 60 mm dish and treated with the indicated concentration of PcOSM for 24, 48 and 72 h. The cells were stained with 20 µM DCFDA for 30 min at 37 °C, trypsinized and neutralized with culture medium and collected in FACS tubes. Flow cytometry analysis was carried out in a flow cytometer (BD FACS Aria II, USA) and the relative DNA content was measured using BD FACSDiva software [55].

### 4.14. Senescence Assay-Beta Galactosidase Detection

Senescence induction of PcOSM was monitored using senescence associated Beta galactosidase (SA-β-gal) detection assay according to the manual provided by the kit (Abcam). Briefly, MDA MB231 cells were grown on a 6-well plate and treated with the indicated concentration of PcOSM for 72 h. The cells were washed with 1 X PBS, treated with fixative solution for 10–15 min at room temperature. Further the cells were washed with PBS and stained overnight at 37 °C in a sealed bag to avoid the pH lowering effect of CO_2_.The cells were observed for the development of blue color and typical morphology of senescent cells using a Nikon microscope.

### 4.15. Cell Cycle Analysis

Cell cycle phase distribution was evaluated by a flow cytometer. Briefly 1 × 10^5^ cells were seeded in a 60 mm dish and treated with indicated concentration of PcOSM for 72 h. The cells were harvested fixed with 70% ethanol and incubated for 1 h at 4 °C. The fixed cells were suspended in PBS and treated with 5 µL of RNase A (5 mg/mL) for 1 h at 37 °C and stained with 2 µL propidium iodide (Sigma-Aldrich) for 10 min at room temperature in dark. The fluorescence of the stained cells was analyzed using flow cytometry (BD FACS Aria II, USA) and the relative percentage of cells distributed in different phases of cell cycle was determined using BD FACS Diva software.

### 4.16. Statistical Analysis

The experiments were repeated thrice with two biological replicates and results are expressed as the mean ± standard deviation. Statistical significance was calculated by unpaired Student’s t test and One Way ANNOVA using Graph Pad prism 6 (Graph Pad software, San Diego, CA, USA). A *p* value < 0.05 was considered statistically significant and for MTT assay, *p* < 0.001.

## 5. Conclusions

The present study reports for the first time the effect of osmotin protein from a wild source (*Piper colubrinum*) in inhibiting *Phytophthora capsici* through induction of ROS in plant. The results suggest an additional role of the osmotin protein in priming plant defense. Interestingly PcOSM could also trigger ROS production in triple negative MDAMB231 cell line, further mediating cellular senescence. These observations are novel and suggest the additional role of osmotin as an inducer of ROS dependent senescence in triple negative breast cancer cell line.

## Data Availability

The data presented in this study are available in the article.

## Sample Availability

Samples of the compounds PcOSM and Osmotin peptide are available from the authors.

## Abbreviations

PcOSM	Full length *Piper colubrinum* Osmotin (693 bop)
ROS	Reactive oxygen species
TBHP	Tert-butyl hydroperoxide
ADIPOR 1	Adiponectin Receptor-1
IPTG	Isopropyl β-d-1-thiogalactopyranoside
DCFDA	2′,7′-Dichlorofluorescein Diacetate
ADIPOQ	Adiponectin
NtOSM	Nicotiana tabacum Osmotin
SA-β-gal	Senescence Associated Beta galactosidase
